# The neural signature of information regularity in temporally extended event sequences

**DOI:** 10.1016/j.neuroimage.2014.12.021

**Published:** 2015-02-15

**Authors:** Jiaxiang Zhang, James B. Rowe

**Affiliations:** a Cognition and Brain Sciences Unit, Medical Research Council, Cambridge CB2 7EF, UK; b Department of Clinical Neurosciences, University of Cambridge, Cambridge CB2 2QQ, UK; c Behavioural and Clinical Neuroscience Institute, Cambridge, CB2 3EB, UK

**Keywords:** fMRI, Information theory, Selection entropy, Trial entropy, Voluntary selection

## Abstract

Statistical regularities exist at different timescales in temporally unfolding event sequences. Recent studies have identified brain regions that are sensitive to the levels of regularity in sensory inputs, enabling the brain to construct a representation of environmental structure and adaptively generate actions or predictions. However, the temporal specificity of the statistical regularity to which the brain responds remains largely unknown. This uncertainty applies to the regularities of sensory inputs as well as instrumental actions. Here, we used fMRI to investigate the neural correlates of regularity in sequences of task events and action selections in a visuomotor choice task. We quantified timescale-dependent regularity measures by calculating Shannon's entropy and surprise from a sliding-window of consecutive task events and actions. Activity in the frontopolar cortex negatively correlated with the entropy in action selection, while activity in the temporoparietal junction, the striatum, and the cerebellum negatively correlated with the entropy in stimulus events at longer timescales. In contrast, activity in the supplementary motor area, the superior frontal gyrus, and the superior parietal lobule was positively correlated with the surprise of each stimulus across different timescales. The results suggest a spatial distribution of regions sensitive to various information regularities according to a temporal hierarchy, which may play a central role in concurrently monitoring the regularity in previous and current events over different timescales to optimize behavioral control in a dynamic environment.

## Highlights

• A sliding-window approach for quantifying trial and selection entropies
• Selection entropy correlates with frontopolar activity at a short timescale.
• A temporal and striatal network is sensitive to task entropy at long timescales.
• Sensorimotor and parietal cortex are sensitive to repetition or surprise in events.

## Introduction

A critical role of cognition is to adaptively control behavior in the face of a dynamically changing environment. Implementing this function requires a neural mechanism that responds to statistical regularities of the environment and its variations, allowing the brain to construct an internal representation of the environmental structure for generating expectations, predicting future events and guiding behavior (Friston et al., 2006; Mumford, 1992; Sokolov et al., 1963). Previous research has demonstrated the ability in infants and adults to identify statistical patterns from temporally extended sequences, and to use this information to form expectations (Amso et al., 2005; Cohen et al., 1990; Curran and Keele, 1993; Gomez, 2002; Kidd et al., 2012). For example, preverbal infants can learn word segments from auditory streams based on the statistical relationships between neighboring syllables (Saffran et al., 1996), and even infants under 3 months can generate expectations of visual stimuli (Canfield and Haith, 1991). Studies of statistical learning in adults further suggest that extracting statistical regularities from sensory inputs facilitates perception (Orbán et al., 2008; Summerfield and Egner, 2009), and this process can occur automatically, without conscious awareness of the underlying patterns (Fiser and Aslin, 2001; Turk-Browne et al., 2005).

Electrophysiological and neuroimaging studies have identified brain regions sensitive to statistical regularities in sensory information, often relying on the response to a single unexpected stimulus as evidence of the prediction arising from detection of regularities in low level or higher order properties of stimuli (Ewbank et al., 2011; Näätänen et al., 1978, 2007; Pazo-Alvarez et al., 2003). Regularities can also be appreciated from temporally extended event sequences, such as the relative frequencies of different events, which formulate macro-scale environmental structures. The predictability or the uncertainty over multiple successive stimuli has been associated with activity of the hippocampus (Harrison et al., 2006; Strange et al., 2005; Turk-Browne et al., 2009, 2010), the prefrontal cortex (Huettel et al., 2002; Kouneiher et al., 2009) and the lateral temporal cortex (Tobia et al., 2012a), even when the statistical features of the sequences are irrelevant to the task (Nastase et al., 2014). These findings advocate the concept of the “proactive” brain (Bar, 2007; Friston et al., 2009), which continuously generates predictions of the relevant future events by extracting information from sensory inputs or internally generated signals (Chiel and Beer, 1997; Friston, 2010).

However, two important issues remain unresolved. First, regularities may exist at various time scales in a dynamic environment (Kiebel et al., 2008; Koechlin et al., 2003). In a tennis game, for example, the player needs to follow and predict the opponent's position to make a successful return (short time scale), and maintain information about the opponent's action sequence in order to make a strategic response (long time scale) (Yamamoto and Gohara, 2000). Although statistical regularities at different time scales can be important in shaping behavior, it is not clear whether different brain regions are sensitive to the regularities at different levels of this temporal hierarchy.

Second, statistical regularity in one's own actions and past choices, is as important as regularity in external sensory inputs when guiding future behavior (Rowe et al., 2010). Regularity in action-selection sequences is also critical for the transition from sensorimotor mapping to skilled motor sequences. Previous studies showed that, over the course of sensorimotor learning, improvements in performance in the early learning stage are based on the order of perceptual events, while the performance in the late learning stage depends on action sequences (Bapi et al., 2000). Further, learnt action sequences can be generalized and transferred to the opposite hand or to other sensory modalities (Cohen et al., 1990; Keele et al., 1995; Willingham et al., 2000). The flexibility of sequence learning requires that the brain does not only learn an action sequence itself, but also the associations between different representations of a sequential structure (sensory events, action events, and selections of actions). While the existing literature focuses on the regularity in sensory inputs, less is known of how the brain encodes the regularity of action selection: most studies have employed paradigms in which actions were either fully determined by sensory inputs (Huettel et al., 2002) or irrelevant to the regularity of sensory information (Nastase et al., 2014).

**Fig. 1:**
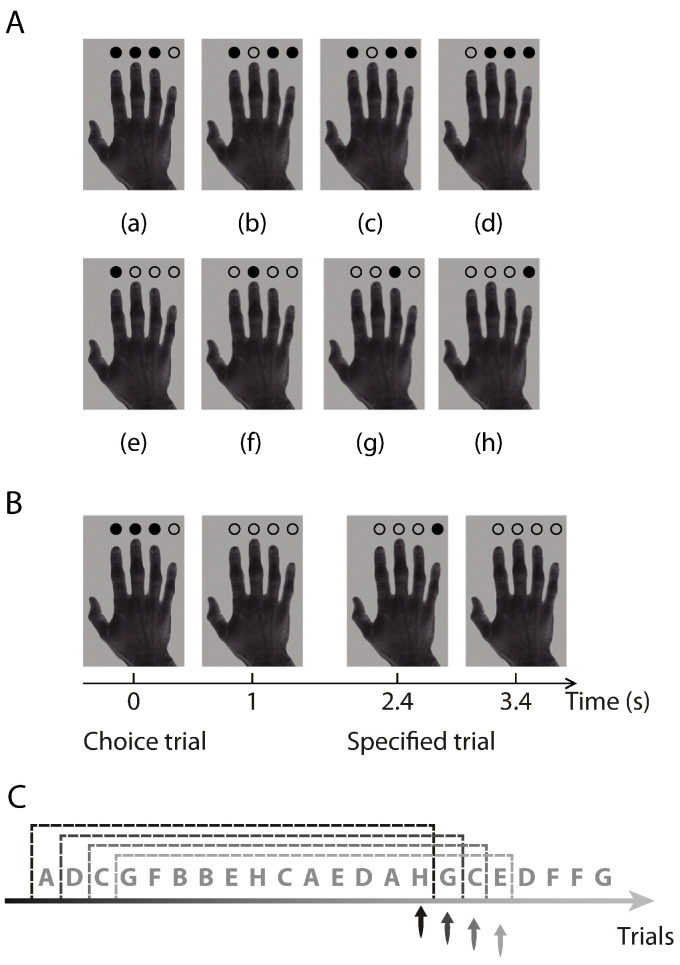
Experiment design and the sliding-window method. (A) Stimuli for the finger-tapping task in the chosen condition (top row) and the specified condition (bottom row). In both chosen and specified trials, permitted actions were indicated by the filled circles and non-permitted actions were indicated by the unfilled circles. (B) Examples of trials. The task stimulus was presented 1000 ms at the beginning of each trial, followed by a 1400 ms interval during which the hand image with four unfilled circles were presented. (C) Entropy measures were calculated in a sliding-window (15-trial length in the illustration) along a sequence of events, and the TE and SE values were assigned to the last trial within each window (as indicated by the arrows). The sliding-window moved forward one trial each time and a new entropy measure was calculated. As such, this approach generated associated TE and SE series from sequences of trial events or actions.

Here, we addressed these two questions by examining fMRI responses to statistical regularities in long sequences of external stimulus events and internally-driven action selections at different time scales. In a visuomotor task (Fig. 1A), participants pressed a button according to a visual stimulus that mapped directly to a single button press in a specified condition; or they voluntarily selected one action from three visually presented alternatives in a chosen condition. In the latter case, the stimuli indicated that subjects should make a response, but not which response to make. By permuting the trial order, and enabling trial-specific response selections, the sequence of ‘internal’ action selections is dissociated from the external trial events. We quantified the statistical regularity by measuring three randomness measures along a sliding window of consecutive stimulus events (i.e., trial entropy and surprise) or selected actions (i.e., selection entropy). By adjusting the length of the sliding window, we identified brain regions sensitive to the randomness measures at different time scales.

Our results demonstrate sensitivity to entropy and surprise: the frontopolar cortex is negatively associated with selection entropy, while the temporoparietal junction, striatum, and cerebellum are sensitive to the trial entropy at a longer time scale. This suggests that the human brain spontaneously monitors statistical regularities in both external events and internal-generated actions, concurrently and at different time scales.

## Materials and methods

### Participants

Sixteen healthy right-handed adults (13 females; age range, 20–39 years; mean age, 26.81 years; s.d. of age, 4.55 years) were recruited from the volunteer panel at the Medical Research Council's Cognition and Brain Sciences Unit, and were paid 20 pounds for their participation. All participants reported normal or corrected-to-normal vision. None had previous experience with the task. Participants responded in 98 ± 1% of the trials on average. The study was approved by the local research ethics committee and was undertaken with the understanding and written consent of each participant.

### Task

Participants performed a visually paced, right-hand finger-tapping task in a single session (Hughes et al., 2013; Rowe et al., 2010; Zhang et al., 2012). Details of the stimuli and task are described in a previous study (Zhang et al., 2012), but the analysis and classification of each trial differs in the current study. Throughout the scanning session, a picture of a right hand was presented on the screen (4.19° × 6.31° visual angle) on a gray background. Four circles (0.39° visual angle) superimposed above the four fingers in the picture, and could be filled or unfilled. The filled circles indicated the fingers that were permitted or required as a response.

There were eight different stimuli under two task conditions (*specified* actions and *chosen* actions) (Fig. 1A). In each of four specified action stimulus, there was one filled circle and three unfilled circles, and participants were required to respond with the finger indicated by the single filled circle. In each of four chosen action stimulus, there were three filled circles and one unfilled circle, and participants could respond with any one of the three fingers indicated by the filled circles. Participants were asked to make a fresh choice on each chosen trial, regardless of what they had done in previous trials. They were not encouraged or discouraged to make or avoid particular actions sequences, nor asked to make “random” choices. For a discussion of cognitive processes underlying such self-ordered choices, see (Hughes et al., 2013; Rowe et al., 2010; Stern et al., 2000; Zhang et al., 2012). The focus of this paper is the temporal regularity structure of the trial and response sequences.

On each trial, the hand picture with filled/unfilled circles were presented for 1000 ms, followed by a 1400 ms interval during which the hand picture with four unfilled circles were presented (Fig. 1B). Participants could make a response at any time after stimulus onset. To improve estimation of the BOLD response differences between task conditions, we used null trials (duration 2400 ms) to vary the SOA while appearing to the subject as the inter-trial screen (Josephs and Henson, 1999; Mechelli et al., 2003).

The experiment session comprised 1008 trials with 50% choice trials, 25% specified trials and 25% null trials. The trials were pseudorandomly intermixed, with no more than four consecutive trials of the same condition. The identity of the task stimulus in each trial further depended on participants' last action: in the chosen condition the option to repeat the last action was available in half of the trials, regardless whether the last action was chosen or specified. Similarly, in the specified condition, the instruction to repeat the last response was presented in half of the trials.

### Quantitative measures of randomness from sliding windows

For each participant's scanning session, there was a finite, discrete set of eight possible stimuli *C_i_* = {*a*, *b*, *c*, *d*, *e*, *f*, *g*, *h*} (*i* = 1, 2, 3, …) (Fig. 1A) and a set of four possible actions *A_i_* = {1, 2, 3, 4}. Note that there were different numbers of chosen and specified trials, and the task stimulus for each condition further related to the participant's previous responses. As a result, the sequences of stimuli and participant's responses could not be completely random.

A sliding-window method was used to quantify the randomness in *C_i_* and *A_i_* (Fig. 1C) (Bollt et al., 2009). For a sliding window with a length of *n* trials, the randomness measures at trial *i* were estimated from the most recent trials within the range [*i* − *n* + 1, *i*], and the randomness measures were assigned to the last trial *i* in the current window (*i* ≥ *n*). The sliding window moved along the *C_i_* and *A_i_* sequences to obtain a continuous measure for all trials *i* (*i ≥ n*) throughout a session.

Three quantitative measures of randomness were estimated. The first randomness measure, hereafter referred to as trial entropy (TE), was defined as the Shannon entropy of all task stimuli within a sliding window. A larger TE value (i.e., high entropy) indicated that the recent task sequence prior to the current trial is more random. The TE at trial *i* with a window length of *n* trials is given by:

$$TE\left(i\right)=H\left(\mathrm{Stimuli}\right)=-\sum_{k=\left\{a,b,c,d,e,f,g,h\right\}}P\left(C_{j}=k\right)\log P\left(C_{j}=k\right),\;\;\left(i-n+1\right)\leq j\leq i\text{,}$$

where *P*(.) is the probability mass function.

The second randomness measure, hereafter referred to as selection entropy (SE), was defined as the conditional entropy of all actions from the chosen condition within a sliding window, given the stimuli of the chosen trials. The SE value indicated the level of randomness of participant's own selection, given the randomness of recent task sequence. The SE at trial *i* with a window length *n* is given by:

$$\begin{array}{l} SE\left(i\right)=H\left(\mathrm{Actions}|\mathrm{Stimuli}=\left\{a,b,c,d\right\}\right)\\ \;=-\sum_{\begin{array}{l} k=\left\{a,b,c,d\right\}\\ m=\left\{1,2,3,4\right\} \end{array}}P\left(A_{j}=m,C_{j}=k\right)\log P\left(A_{j}=m|C_{j}=k\right),\left(i-n+1\right)\leq j\leq i\text{.} \end{array}$$

The third measure is the “surprise” (SUP) (Strange et al., 2005), which quantifies the amount of information conveyed by the current stimulus in the context of recent events in a specified window. If the stimulus in the current trial *i* is *k*, (*k* = {a, b, c, d, e, f, g, h}), the SUP with a window length *n* is given by:

$$SUP\left(i\right)=-\log P\left(C_{j}=k\right),\;\left(i-n+1\right)\leq j\leq i\text{.}$$

We assumed that all the measures are sustained state representations of the randomness based on recent trials, and therefore TE, SE, and SUP are all modeled on a trial-by-trial basis, regardless whether the current trial is a chosen or specified trial. In particular, we modeled the SE based on all recent chosen trials even when the current trial is a specified trial, although participants did not make selections in specified trials and their responses were fully determined by the task stimulus. By changing the position and size of the sliding-windows, we can quantify the three randomness measures and their fluctuations at different temporal scales. For each participant, TE, SE and SUP values were estimated at six different sliding-window lengths (25–50 trials, step size 5 trials). The randomness measures were used as parametric modulators in analysis of fMRI data.

### Imaging data acquisition

A Siemens Tim Trio 3 T scanner (Siemens Medical Systems, Germany) with 12-channel head coil was used to acquire BOLD sensitive T2* weighted echo-planar images (TR = 2000 ms, TE = 30 ms, FA = 78 degrees, 32 × 3mm slices, in-plane resolution 3 × 3 mm with slice separation 0.75 mm, sequential descending order). One thousand three hundred volumes were acquired in a single session and the first six volumes were discarded to allow for steady-state magnetization. Participants also underwent high resolution magnetization prepared rapid gradient echo scanning (MP-RAGE: TR = 2250 ms, TE = 2.99 ms, FA = 9 degrees, IT = 900 ms, 256 × 256 × 192 isotropic 1 mm voxels). Visual stimuli were presented by using Matlab 7.8 (Mathworks, Natick, MA) and the Psychtoolbox-3 (www.psychtoolbox.org), and were displayed onto a screen using a Christie video projector with a resolution of 1024 × 768 and a refresh rate of 60 Hz.

### fMRI data preprocessing

MRI data were processed using SPM8 (www.fil.ion.ucl.ac.uk/spm). fMRI data were converted from DICOM to NIFTII format, spatially realigned to the first image, and corrected for acquisition delay with references to the middle slice. The mean fMRI and MP-RAGE images were coregistered using mutual information, and the MP-RAGE image was segmented and normalized to the Montreal Neurological Institute (MNI) T1 template by linear and non-linear deformations. The normalization parameters were applied to all spatiotemporally realigned functional images, and normalized images were resampled to 3 × 3 × 3 mm before smoothing with an isotropic Gaussian kernel with full-width half-maximum of 8 mm.

### fMRI data analysis

For each sliding-window length, a first-level general linear model (GLM) included five regressors. The first regressor represented onsets of stimulus presentation in all trials. The second regressor contrasted the choice and the specified trials (consisting + 1 and − 1), modeling the differences between the two task conditions (Rowe et al., 2010). The three additional parametric modulators represented the TE, SUP and SE values across trials, estimated from the sliding-window method and mean-corrected to the entire session. Response times from individual trials were entered as a regressor of no interest. All parametric regressors were mean-corrected and orthogonalized with respect to their previous regressor by using the Gram–Schmidt orthogonalization procedure in SPM.^1^ Therefore, the SUP regressor was orthogonalized with respect to TE to account for the variance that cannot be attributed by the TE values and the SE regressor was orthogonalized with respect to SUP and TE because SE is conditioned on trial information by definition. Two additional regressors were included to model the error trials in the chosen and specified conditions (i.e., trials with invalid responses or trials without responses, which accounted for 4.54% of all trials). Imaging volumes within the first sliding-window of a scan session were removed from analysis, because the randomness measures were not available prior to the first window. Six rigid-body motion correction parameters were included as nuisance covariates. Regressors were convolved with a canonical hemodynamic response function, and the data were high-pass filtered with a frequency cutoff at 400 s.

First-level contrast images of TE, SE and SUP from the six sliding-window lengths were entered into three second-level ANOVAs, adjusted for non-sphericity with dependence between measures and unequal variance. A liberal threshold was firstly used to localize the effect of each randomness measure averaged across the six sliding-window lengths (*p* < 0.001 uncorrected and cluster of 50 voxels or more). Regions of interest (ROIs) were then defined at the peaks of the significant clusters (with 8 mm radius) using the MarsBar toolbox (http://marsbar.sourceforge.net). Averaged regional BOLD response was tested for differences between different window lengths by using repeated-measures ANOVA. For each analysis, results were reported as statistically significant for *p* < 0.05, corrected for FDR using the Benjamini–Hochberg procedure (Benjamini and Hochberg, 1995).

Next, we identified supra-threshold activities associated with the randomness measures at each individual window-length in a post-hoc exploratory analysis. For each sliding-window length, five first-level contrast images (the effect of stimulus presentation in all trials, the three randomness measures, and the contrast between choice and specified conditions) from each participant were entered into a second-level analysis, and statistical parametric maps were generated for each effect of interest. Results in the statistical parametric maps were reported at a cluster extent threshold corrected for multiple comparisons using Gaussian random field theory (*p* < 0.05, FDR) with a conventional voxelwise threshold (*p* < 0.001 uncorrected) (Chumbley and Friston, 2009).

## Results

**Fig. 2:**
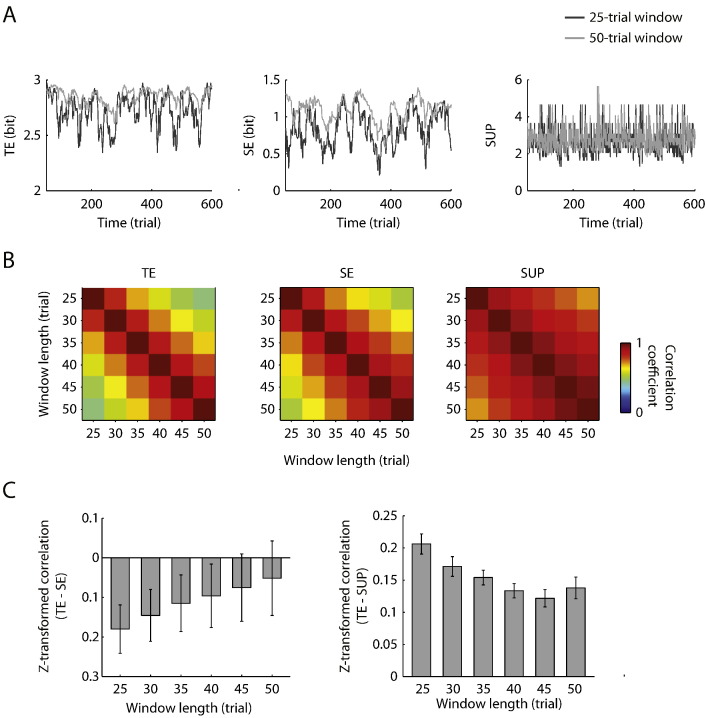
Randomness measures. (A) TE (left), SE (middle), and SUP (right) values at two different window lengths (25-trial and 50-trial) from one single participant. (B) Mean correlation coefficients of the randomness measures from different window lengths. For each randomness measure, a Pearson correlation matrix was calculated between two of six possible window lengths and averaged across participants. (C) The mean Fisher z-transformed correlations between TE and SE (left) and between TE and SUP (right). Error bars represent 95% bootstrapping confidence interval.

We estimated three types of randomness in a right-hand finger-tapping task: trial entropy (TE) quantifying the randomness of external stimulus, selection entropy (SE) quantifying the randomness of action selection, and surprise (SUP) quantifying the information content of the current stimulus. For each participant, the TE, SE and SUP values were calculated from a sliding window of consecutive trials, and were assigned to the last trial in the window (Fig. 1C). By incrementally sliding the window along the entire trial sequence in each scanning session, we obtained TE, SE and SUP series that continue and evolve over time. All the three randomness series from individual participants exhibited fluctuations over time (see Fig. 2A for the randomness series from an individual participant). Such variances in the randomness series could be used to inform the analysis of fMRI data. Below, we reported the characteristic properties of the randomness series, and then identified brain regions that were associated with the different measures at different time scales.

### Randomness in trial and selection sequences

We examined the similarity of each randomness measure at different temporal scales by calculating Pearson's correlations of each randomness series estimated from six window lengths (25 to 50-trial windows with an incremental step of 5 trials). The randomness measures from two similar window lengths were strongly correlated with each other (Fig. 2B). This is expected because any two sliding windows with similar length share a proportion of information. For example, compared with a 25-trial window, a 30-trial window contains five new trials and shares the rest of the data with the 25-trial window. As the difference of the two windows' length increases, the amount of shared information decreases and a randomness measure from the two windows become less correlated. We compared the Fisher z-transformed correlations of SE, TE and SUP between the two most distinct window lengths (25 and 50 trials). A nonparametric analysis of variance for repeated measures (Friedman's test) showed significant higher correlation in SUP than that in SE and TE (*p* < 0.00001). In other words, window length had larger effects on entropies (TE and SE) than on surprise.

We then examined the relations between TE and the other two randomness measures (Fig. 2C). SUP was positively correlated with TE at all window lengths (*p* < 0.001, one-sample Wilcoxon signed-rank test) and the correlations were higher for small windows than for large windows (*p* < 0.00001, Friedman's test). Further, there was also a significant main effect of window length on the correlations between TE and SE (*p* < 0.00001, Friedman's test), and the SE was negatively correlated with TE across participants at small windows (25-trial window, *p* < 0.001; 30-trial window, *p* < 0.01; 35-trial window, *p* < 0.05; 40-trial window, *p* < 0.05, one-sample Wilcoxon signed-rank test), but the correlations were not significant at larger windows (45-trial window, *p* = 0.08; 50-trial window, *p* = 0.18).

To test whether the randomness measures could modulate response time (RT) (e.g., Jamieson and Mewhort, 2009), we conducted a within-subject GLM analysis using single-trial RT as the dependent variable and the three randomness measures as the independent variables. Coefficient estimates from individual participants were then entered into a second-level analysis. No significant effect of TE, SE and SUP on the RT was observed at any window length (supplementary fig. S1). The lack of relationship between the randomness measures and RT implies that our fMRI analysis was not confounded by trial-by-trial variations in RT.

To test whether the randomness measures could modulate response time (RT) (e.g., Jamieson and Mewhort, 2009), we conducted a within-subject GLM analysis using single-trial RT as the dependent variable and the three randomness measures as the independent variables. Coefficient estimates from individual participants were then entered into a second-level analysis. No significant effect of TE, SE and SUP on the RT was observed at any window length (Supplementary Fig. S1). The lack of relationship between the randomness measures and RT implies that our fMRI analysis was not confounded by trial-by-trial variations in RT.

### fMRI BOLD responses associated with randomness

**Fig. 3:**
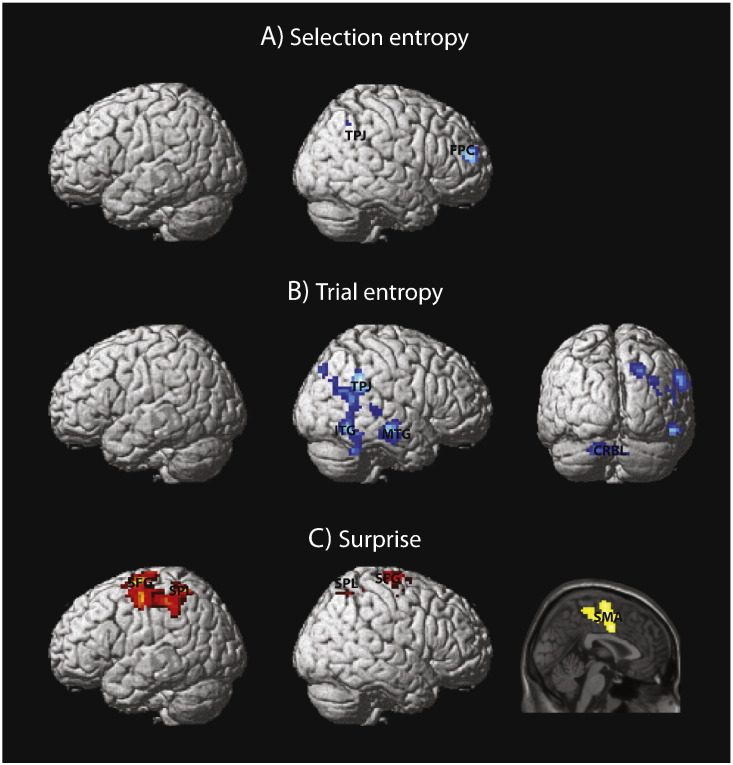
Brain regions showing significant responses to (A) SE, (B) TE and (C) SUP averaged across different window lengths (illustrated at *p* < 0.001 uncorrected, cluster size > 50 voxels). Peak MNI coordinates in (A) FPC (39, 53, 19), TPJ (30, − 58,43); (B) CRBL (− 15, − 73, − 29), MTG (63, − 19, − 14), ITG (48, − 55, − 8), TPJ (57, − 46, 31); and (C) SFG (left, − 27, − 7, 64; right, 15, − 16, 67), SMA (6, − 10, 58), SPL (left, − 27, − 52, 55; right 21, − 55, 55). The cluster in (B) comprising the TPJ and ITG survived whole brain cluster-extent correction (*p* < 0.05), and the cluster in (C) also survived whole brain cluster-extent correction (*p* < 0.05).

We used individual participant's randomness series to inform the models of BOLD responses. A whole-brain random-effect analysis showed brain regions that were associated with SE, TE, and SUP when averaged across the six window lengths (Fig. 3). SE negatively correlated with BOLD responses in the right frontopolar cortex (FPC) and the right temporal-parietal junction (TPJ) (*p* < 0.001 uncorrected, cluster size > 50 voxels). TE negatively correlated with the activity in the right TPJ, the right middle temporal gyrus (MTG), the right inferior temporal gyrus (ITG), and the right cerebellum. In contrast, SUP was positively associated with bilateral activity in the supplementary motor area (SMA), the superior frontal gyrus (SFG), and the superior parietal lobule (SPL). No significant activation was observed from reverse contrasts testing for a positive BOLD correlation to SE or TE, or a negative correlation to SUP. In summary, these regions showed increased BOLD responses when recent stimulus or action sequences are more regular (i.e., low trial and selection entropies), or when current events had higher unpredictability (i.e., high surprise).

**Fig. 4:**
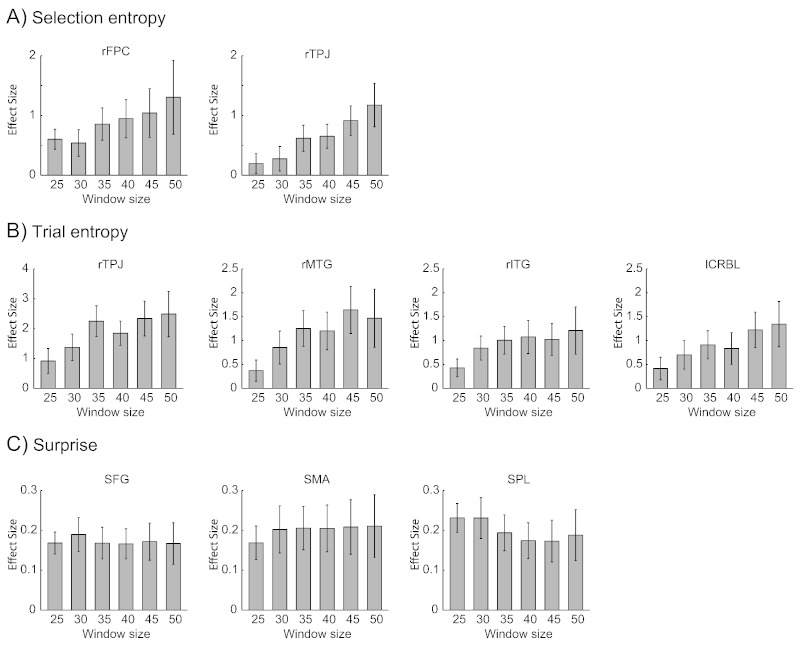
The regional effect size (beta) of BOLD response to the (A) SE, (B) TE and (C) SUP at different window lengths. The regions of interest were defined at the peaks of the significant clusters (with 8 mm radius) that showed averaged effect across all windows for each outcome measure (Fig. 3). The bars denote the averaged response in the ROIs. Error bars denote standard errors across participants. (A) and (B) showed the absolute value of the effect size because the correlation between the BOLD response and entropy measures are negative.

The regions of interest (ROI) were defined as spheres of 8 mm radius centered on peak coordinates of randomness-related activation averaged across the six window lengths. All ROIs except FPC showed increased BOLD response to stimulus onset (Supplementary Fig. S2). Therefore the negative BOLD-entropy correlations suggest increased activation with more ordered sequence. We then examined whether the BOLD-randomness associations depended on the length of the sliding-window in each ROI (Fig. 4). For SE, a repeated-measures ANOVA showed a significant effect of window length in the TPJ (*F*(5,75) = 6.97, *p* < 0.001, FDR corrected) but not in the FPC (*F*(5,75) = 1.71, *p* = 0.14). For TE, there was a significant effect of window length in the TPJ (*F*(5,75) = 2.82, *p* < 0.05, FDR corrected), MTG (*F*(5,75) = 3.67, *p* < 0.01, FDR corrected), and cerebellum (*F*(5,75) = 3.44, *p* < 0.01, FDR corrected), but not in the ITG (*F*(5,75) = 1.67, *p =* 0.15). No significant effect of window length was observed on the BOLD response to the SUP (SMA, *F*(5,75) = 0.42, *p* = 0.84; SFG, *F*(5,75) = 0.28, *p* = 0.92; SPL, *F*(5,75) = 1.36, *p* = 0.25). Post-hoc analysis on the regions with a significant main effect of window length showed that the SE effect at 50-trial window was larger than that at 25-trial window in the TPJ (*t*(15) = 3.04, *p* < 0.01). The TE effect at 50-trial window was larger than that at 25-trial window in the MTG (*t*(15) = 2.14, *p* < 0.01) and cerebellum (t(15) = 2.30, *p* < 0.05), and the difference was marginal in TPJ (*t*(15) = 2.10, *p* = 0.05). supplementary Fig. S4 shows how the BOLD response negatively scales with SE and TE at 25-trial and 50-trial windows.

The regions of interest (ROI) were defined as spheres of 8 mm radius centered on peak coordinates of randomness-related activation averaged across the six window lengths. All ROIs except FPC showed increased BOLD response to stimulus onset (Supplementary Fig. S2). Therefore the negative BOLD-entropy correlations suggest increased activation with more ordered sequence. We then examined whether the BOLD-randomness associations depended on the length of the sliding-window in each ROI (Fig. 4). For SE, a repeated-measures ANOVA showed a significant effect of window length in the TPJ (*F*(5,75) = 6.97, *p* < 0.001, FDR corrected) but not in the FPC (*F*(5,75) = 1.71, *p* = 0.14). For TE, there was a significant effect of window length in the TPJ (*F*(5,75) = 2.82, *p* < 0.05, FDR corrected), MTG (*F*(5,75) = 3.67, *p* < 0.01, FDR corrected), and cerebellum (*F*(5,75) = 3.44, *p* < 0.01, FDR corrected), but not in the ITG (*F*(5,75) = 1.67, *p =* 0.15). No significant effect of window length was observed on the BOLD response to the SUP (SMA, *F*(5,75) = 0.42, *p* = 0.84; SFG, *F*(5,75) = 0.28, *p* = 0.92; SPL, *F*(5,75) = 1.36, *p* = 0.25). Post-hoc analysis on the regions with a significant main effect of window length showed that the SE effect at 50-trial window was larger than that at 25-trial window in the TPJ (*t*(15) = 3.04, *p* < 0.01). The TE effect at 50-trial window was larger than that at 25-trial window in the MTG (*t*(15) = 2.14, *p* < 0.01) and cerebellum (t(15) = 2.30, *p* < 0.05), and the difference was marginal in TPJ (*t*(15) = 2.10, *p* = 0.05). Supplementary Fig. S4 shows how the BOLD response negatively scales with SE and TE at 25-trial and 50-trial windows.

The above analysis suggested that certain brain regions with averaged effects across different window lengths were differentially sensitive to SE or TE at different temporal scales. But this analysis cannot test whether some regions significantly respond to randomness measures at individual window length. Therefore, in an exploratory analysis, we examined supra-threshold BOLD responses to the SE and TE, separately for each of the six window lengths. The SE negatively correlated with the activity in bilateral frontopolar cortex (FPC) at the 25-trial window (*p* < 0.05 cluster-corrected, Table 1). No significant activation was observed for the SE at other longer windows. To determine whether the effect of SE was specific to the short window, we exclusively masked the results of the 25-trial window with the results of the longest 50-trial window at a threshold of *p* < 0.05 (uncorrected). The bilateral FPC survived this disjunction analysis at a lower threshold (*p* < 0.001 uncorrected, cluster size > 50 voxels). The results from the disjunction tests did not survive cluster-extent corrections for multiple comparisons.

TE negatively correlated with the activation in the TPJ, the temporal cortex, the frontopolar cortex, and the cerebellum at short to medium window lengths (30, 35 and 40 trials) (*p* < 0.05 cluster-corrected, Table 1). At longer windows (45 and 50 trials), TE also negatively correlated with the activation in the striatum and the anterior cingulate cortex. Exclusive masking of the results of the 50-trial window by the results of the 25-trial window at a threshold of *p* < 0.05 (uncorrected), revealed that only the striatum survived the disjunction analysis (*p* < 0.001 uncorrected, cluster size > 50 voxels), suggesting that the subcortical response to TE may be specific to longer windows.

### Repetition versus alternation of stimuli and responses

We have shown that BOLD responses in different brain regions negatively associated with TE and SE from a sequence of previous trials. We tested whether these effects could be driven by the most recent stimulus (i.e., switching or maintaining task stimuli) or action (ie. same versus different action). This would predict increased BOLD response in stimulus repetition or action repetition (low entropy) compared with alternation (high entropy) in two consecutive trials (see Tobia et al., 2012a,b, for a detailed discussion). We therefore directly contrasted task trials with repetition and alternation of stimulus presentation (regardless participant's actual actions), and task trials with repetition and alternation of actions (regardless which task stimulus was presented).

**Fig. 5:**
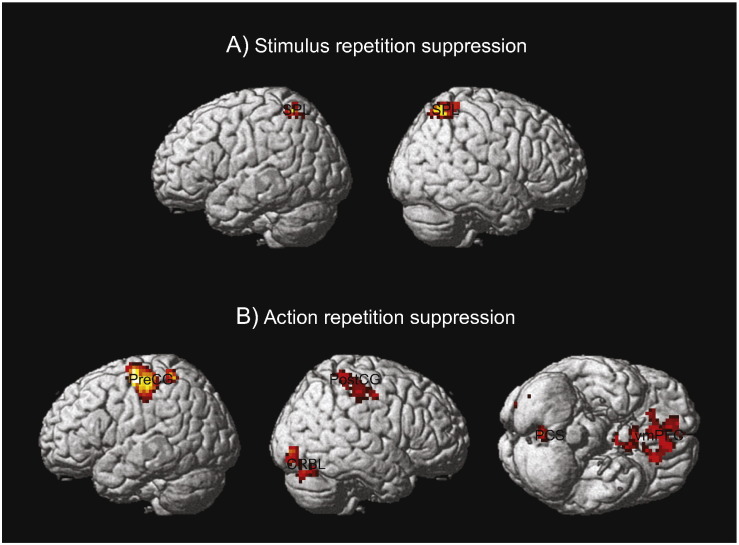
Brain regions showing significant repetition suppression effects in (A) stimulus repetition and (B) action repetition (*p <* 0.05, cluster level corrected). Peak MNI coordinates in (A) SPL (left: − 18, − 58, 58; right: 21, − 58, 58) and (B) precentral gyrus (preCG, − 36, − 16, 58), postcentral gyrus (postCG, 36, − 42, 64), ventromedial frontal (vmPFC, 0, 14, − 2), precuneus (PCS, 3, 55, − 13), CRBL (15, − 73, 23).

No voxels showed significant activity in repeating versus switching task stimuli or actions. This result suggested that the entropy effects cannot be simply driven by the most recent event. Instead, there were repetition suppression effects (Fig. 5). Switching compared with repeating *stimulus presentation* was associated with increased activation in the superior parietal cortex (*p* < 0.05 cluster-corrected). Switching compared with repeating *actions* was associated with increased activation in the sensorimotor cortex, the ventromedial frontal cortex, and the cerebellum.

## Discussion

This study has identified anatomical specificity in the neural correlates of three types of information theoretic measures: the entropy of stimulus sequences, the entropy of action selections, and the surprise of current stimulus. In addition, there is evidence for temporal specificity in trial entropy, and weak evidence for temporal specificity of selection entropy in some regions. At longer window lengths, an extensive network including the TPJ, striatum, and cerebellum was associated with TE. The activity in the FPC correlated with the SE at a short window length (25 trials: although the effect of window length was not significant). In contrast, activity in the sensorimotor cortex was associated with the surprise across all window lengths. Together, these results provide new insights into the fundamental processes of monitoring and prediction, demonstrating cortical and subcortical sensitivity to regularity or uncertainty, in sensory inputs and action selections over short and long timescales.

Previous studies have linked information theoretic indices of randomness in sensory and motor sequences to brain activation, including Shannon's entropy, mutual information, conditional entropy, and surprise (Bischoff-Grethe et al., 2000, 2001; Harrison et al., 2006, 2011; Nastase et al., 2014; Strange et al., 2005; Tobia et al., 2012a,b), but give different interpretations of their functional significance. One interpretation is that a relation between BOLD responses and statistical regularity of the environment indicates the involvement of brain regions in the computation of the randomness measures. For example, hippocampus and paralimbic structures have been proposed to encode the uncertainty of the environment based on a Bayesian observer model (Geisler, 1989). This interpretation is supported by positive correlations between hippocampal activity and Shannon's entropy in visual stimulus streams (Strange et al., 2005), and the mutual information in consecutive events in a sequential reaction time task (Harrison et al., 2006).

However, a BOLD-randomness relation alone does not imply the encoding of entropy *per se*, but can also be interpreted as the presence of other cognitive processes that are sensitive to entropy measures (Tobia et al., 2012b). A positive correlation between randomness and BOLD could indicate predictive processing of error signals, because larger prediction errors are expected in more random sequences within a predictive coding perspective (Bubic et al., 2010; Rao and Ballard, 1999). Conversely, a negative correlation could occur if a region is sensitive to the degree of predictability of the current event given the knowledge of recent events (Bischoff-Grethe et al., 2001; Tobia et al., 2012b).

We found systematic negative correlations between BOLD response and the two entropy measures (TE and SE) across different brain regions, and positive correlations between BOLD response and surprise. This extends previous studies showing negative BOLD-entropy relations under the presence of visual or auditory sequences (Nastase et al., 2014; Tobia et al., 2012a,b) and positive BOLD-surprise relations in serial reaction time paradigm (Strange et al., 2005). A more recent study also reported that BOLD response positively correlated with the surprise of events when participants observing another person's action sequences (Ahlheim et al., 2014).

Several cognitive processes could account for the direction of the BOLD-entropy correlations in our study. For example, it is consistent with the possibility of an online monitoring process that constantly evaluates statistical regularities from recent event sequence. This monitoring process would invoke increased BOLD response when the observed frequency of different events deviates from a random sequence (i.e., low entropy state), and thereby allow the system to generate predictions of future events. Alternatively, the effect of selection entropy could relate to conscious attention to self-initiated, systematic choice sequences which have low entropy. Interestingly, frontal-lobe damage is often associated with inflexible and preservative behavior (Burgess and Shallice, 1996; Duncan, 1986), suggesting that the prefrontal cortex is essential to switch behavior at different levels of randomness (Rowe et al., 2010). In order to distinguish the cognitive processes underlying the BOLD-entropy associations, future experiments should investigate how voluntary action selection can be affected by previous action sequences.

It is possible that participants paid more (or less) attention to the task over the course of the experiment, but a lapse of attention on the task is less likely to account for our results, because BOLD response to the entropy in sensory inputs has been shown in both active tasks (instruct to explicitly monitor regularity) and passive tasks (instruct to ignore the sensory input and perform an orthogonal task) (Tobia et al., 2012a). Furthermore, the lack of association between RT and selection entropy does not support that entropy fluctuation relates to a lapse of attention, which may lead to increased response time. The negative BOLD-entropy correlation also makes it unlikely that our results are simply driven by neural repetition suppression (Grill-Spector et al., 2006). The repeated occurrence of a stimulus or an action invokes reduced neural response in specific sensory or motor regions (Hamilton and Grafton, 2009; Majdandzic et al., 2009; Summerfield et al., 2008), and indeed we observed repetition-related BOLD signal reductions in the parietal cortex for stimulus repetition and in the sensorimotor cortex for action repetition. However, more repetitions of a task stimulus or an action within a sliding-window also introduce unequal probabilities of events, and lead to a lower entropy value. As a result, neural repetition suppression effects alone would be expected to lead to a positive BOLD-entropy relation, which is not supported by our data.

Many studies on the neural correlates of randomness have calculated information theoretic indices based on all previous events (Bestmann et al., 2008; Harrison et al., 2006; Mars et al., 2008; Strange et al., 2005) or on a fixed block of events (Bischoff-Grethe et al., 2000, 2001; Tobia et al., 2012a). These approaches introduce implicit assumptions of temporal scales on randomness measures and may, in turn, impose constraints to imaging results. For example, if a brain region responds to randomness measures at a specific time scale (e.g., Ostwald et al., 2012), calculating entropy from all previous events would be less sensitive to detect such an effect.

The current study did not make *a priori* assumptions about the temporal scale, but considered entropy measures at multiple window lengths. In order to get meaningful entropy measures, our analysis was focused at a range of window lengths from 25 to 50 trials, which is also in line with previous studies on the neural representations of statistical information over long temporally-extended sequences of events (Harrison et al., 2006; Strange et al., 2005; Tobia et al., 2012a). This method revealed that posterior cortical and subcortical regions were more sensitive to TE from a longer window. This contrasts with a temporal discounting model of a serial response task (Harrison et al., 2011) which gives greater weight to recent events. However, Harrison et al. used a demanding probabilistic visuomotor association task as opposed to the current deterministic task (with direct stimulus–response mapping and chosen actions). These task differences may underlie the differential effects of window length.

Our study provides new insights into the hierarchy of brain regions responding to statistical regularities in a dynamic environment. It has been proposed that the lateral frontal cortex embodies a rostro-caudal hierarchy that is sensitive to different time scales of environmental dynamics (Badre, 2008; Koechlin and Hyafil, 2007; Koechlin et al., 2003). In this model, more caudal frontal regions engage faster dynamics and more rostral frontal regions respond to dynamic changes at a larger time scale (Badre, 2008; Botvinick, 2008; Fuster, 2004). Our results on selection entropies suggested that the frontopolar cortex may be sensitive to selection entropy changes at a relatively short time scale (25 trials). However, our results on trial entropies also suggest a spatially distributed temporal hierarchy elsewhere. At more extended temporal scales (> 35 trials), brain activations associated with statistical regularities include temporal and parietal cortex. Therefore, some brain regions are more affected by recent information than others.

We propose that this temporal-dependent BOLD-entropy association serves two functions. First, if the information of temporal structure from more distant events mainly affects neural activities in parietal and temporal regions, the prefrontal cortex is available to monitor more recent events as a priority within a critical window (Huettel et al., 2002). Second, both recent and distant events could be important in a dynamic environment, but with different consequences for a current response mediated by cognitive processes on the monitored past events. The areas associated with TE over a longer time scale in our study (i.e., striatum and TPJ) are implicated in cognitive processes that integrate information and experience from distant past actions, such as sensorimotor learning (Bischoff-Grethe et al., 2000, 2001), reward-based learning (Gottfried et al., 2003; O'Doherty et al., 2004), habituation (Graybiel, 2008; Yin and Knowlton, 2006), and the sense of agency (Chambon et al., 2012; Farrer et al., 2008). Conversely, impairments of the right angular gyrus have been associated with neglect of actions (Mort et al., 2003).

In the current study, hippocampal activity did not correlate with entropy or surprise. This is in line with recent studies showing that hippocampus is not sensitive to entropy or uncertainty changes in temporally extended event sequences (Nastase et al., 2014; Tobia et al., 2012a). To the contrary, it is suggested that the hippocampus is involved in uncertainty processing in statistical learning (Davis et al., 2012) and serial choice tasks (Strange et al., 2005; Harrison et al., 2006; but see Harrison et al., 2011). We think this apparent distinction in the literature is at least partly due to different task features employed in previous studies. Studies reporting the involvement of hippocampus in the coding of entropy often required participants to choose or match between targets and other items (e.g., Turk-Browne et al., 2009), which actively engage the hippocampal episodic memory system.

In our paradigm, participants chose one of three permitted actions in the chosen condition. Such ‘voluntary’ action selection has been shown to involve the formation of internal intentions (Haggard, 2008; Libet et al., 1983) and engage the frontoparietal network (Forstmann et al., 2006; Haynes et al., 2007; Rowe et al., 2008, 2010; Soon et al., 2008; Walton et al., 2004; Zhang et al., 2012). Because the actions were not associated with differential outcomes in our task (i.e., there were no correct or incorrect actions), participant's decisions were probably not determined by differential expected rewards or sensory stimuli. Such a design allows one to separately quantify the randomness in sequences of actions and sequences of trial events. It also extends previous work focusing only on the degree of order in the sensory inputs, in which behavioral responses are either fully determined by sensory stimuli (Bischoff-Grethe et al., 2000, 2001; Harrison et al., 2011; Strange et al., 2005) or irrelevant to the stimulus sequence of interest (Nastase et al., 2014; Tobia et al., 2012a,b).

It is also of interest that the same frontopolar cortical region is sensitive to randomness in both action selections and trial events. This accommodates a normative account of action and perception based on the free-energy principle by which both action and perception minimize the long-term averaged surprise (note that this function is not the same as SUP as defined in this study) (Friston, 2010; Friston et al., 2010). However, perception and action occur within a hierarchy of beliefs, prediction and prediction errors. Layers of this hierarchy can operate over different time scales, reflecting at one extreme an agent's personality and psychological traits through to transient or local events represented by the SUP function in the current study (Adams et al., 2013; Edwards et al., 2012). Participants cannot *act* to alter SUP or TE, which are determined by the experimenter, but they could nonetheless alter their beliefs based on recent experience so as to minimize their free-energy. A common region might subserve this operation by providing information (or beliefs) about recent actions and events (Schubotz, 2007). However, the voluntary actions are not completely independent from trial events in our study (i.e., at least one out of four actions was not permitted in each trial) and the chosen trials were intermixed with specified trials. This could partly explain the lack of BOLD-randomness relations to selection entropy at longer window: it is difficult to monitor recent action selections in the presence of other actions specified by intervening task stimuli. Future studies on endogenously generated action sequences that are not constrained by task stimuli will complement our findings here and provide further evidence on brain's response to action regularities.

There are several limitations to the current study. First, we examined three different randomness measures. However, other types of randomness indices are available (e.g., Harrison et al., 2011; Kidd et al., 2012). For example, TE was defined as the frequency of the eight different stimuli, which relates to previous studies on the entropy of sensory inputs (Tobia et al., 2012b). One can also study an entropy measure on the relative frequency of chosen and specified trials. However, our current design is underpowered for this analysis because the ratio of chosen and specified trials had low variance across different windows. Future study could systematically permute the distribution of different trial types and examine how the brain responds to the randomness of trial types (not trial stimuli).

Second, we conducted separate regression models at different window lengths and used disjunction tests as a complementary analysis to the ROI analysis to estimate the temporal specific of BOLD-randomness associations. However, disjunction tests only illustrate regions that are activated in one contrast (e.g., 25-trial window) and not by the other contrast (e.g., 50-trial window), but do not necessarily support the hypothesis that the BOLD response between two window lengths is different. An alternative approach is to include all entropy measures from different window lengths into a single first-level model. However, this single-model approach would be sub-optimal for the current study, because the entropy measures from different window lengths were estimated from the same event sequence and correlated with each other.

Third, although the averaged activation across different windows reached corrected significance in most ROIs, a few clusters only passed a more lenient uncorrected cluster threshold *k >* 50 (i.e., FPC, cerebellum, and MTG). Nevertheless, this is in line with several previous studies, which showed smaller effects for the BOLD-randomness association (Strange et al., 2005; Tobia et al., 2012b). The significant results at individual window size (Table 1) suggest that the effects in these clusters are more robust than assumed by the lenient threshold used to localize the ROIs. A combination of uncorrected voxelwise and cluster threshold produces a desire balance between type I and II error rates, in particular for moderate effects (Lieberman and Cunningham, 2009). A future study should aim to replicate and extend our findings in an independent dataset.

In summary, we investigated neural responses to regularity structures from past trial events and action sequences by using a parametric sliding-window approach for fMRI. Our study reveals the brain regions with differential sensitivity to statistical regularities in temporally extended event sequences. We suggest that these multiple systems concurrently monitor changes in external environment and internally generated responses, enabling adaptive behavior to be based on both recent and distant information.

The following are the supplementary data related to this article.

Fig. S1 Generalized linear model of RT. Within-subject GLMs included TE, SE and SUP values as predictors of single-trial RT. Coefficient estimates from individual participants were averaged. Error bars represent the standard errors across the participants. No significant effect of TE, SE or SUP on the RT at any window length (p > 0.09, Wilcoxon signed-rank test).

Fig. S2 Regional BOLD response associated with trial onset. The ROIs were defined as regions with significant associations to (A) SE, (B) TE and (C) SUP as in Fig. 4. Error bars denote standard errors across participants. All ROIs had increased activity at trial onset(*t*(15) > 3.53, *p* < 0.01) except rFPO (*t*(15) = 2.02, *p* = 0.06, one-sample t-test).

Fig. S3 Brain regions showing significant responses to (A) SE, (B) TE and (C) SUP averaged across different window lengths (p<0.001 uncorrected, cluster size k>50 voxels). The model and analysis were the same as in Figure 3. The parametric regressors were only orthogonalized with respect to the trial onset regressor, and serial orthogonalization was not applied. The table below lists the statistics at peek coordinates from the model with serial orthogonalization (Figure 3) and the statistics at the same coordinates from the model without serial orthogonalization.

Fig. S4 Effect size plots for (A) the SE effect, and (B) the TE effect at 25-trial (blue) and 50-trial (red) window sizes. Data plotted separately for trials in which the SE or TE values were low and high in five bins (20/40/60/80/100 percentile of value range). The solid points are individual subjects' data and the open points are averaged effect size across subjects.

Supplementary data to this article can be found online at http://dx.doi.org/10.1016/j.neuroimage.2014.12.021.

## Figures and Tables

**Table 1:** Regions associated with TE and SE at different window lengths. Statistics (*p* < 0.05 cluster-corrected with *p* < 0.001 voxelwise threshold) and peak coordinates reported in MNI space (mm). Note that the separate contrasts at multiple window lengths are not independent (see Discussion).

	Window length (trials)	Region	*t*	Cluster-extent threshold	*x*	*y*	*z*
SE	25	Frontopolar cortex	− 5.05	111	− 27	53	19
			− 4.29		24	59	10
TE	30	Inferior temporal gyrus	− 4.53	203	− 48	− 34	− 8
	35	Frontopolar cortex	− 4.61	122	30	56	− 5
		Middle temporal gyrus	− 5.25		63	− 22	− 14
		Temporoparietal junction	− 5.25		60	− 46	34
		Cerebellum	− 4.34		− 15	− 73	− 29
	40	Temporoparietal junction	− 4.30	194	54	− 61	22
		Middle temporal gyrus	− 3.86		57	− 19	− 11
	45	Caudate	− 4.88	108	12	17	− 5
			− 5.17		− 12	14	− 8
		Anterior cingulate	− 4.53		6	35	− 8
			− 4.22		− 6	32	− 8
		Temporoparietal junction	− 4.56		54	− 52	25
			− 4.72		− 39	− 58	31
		Middle temporal gyrus	− 4.77		63	− 10	− 17
		Cerebellum	− 5.00		15	− 73	− 32
			− 4.65		− 15	− 73	− 29
	50	Temporoparietal junction	− 4.99	110	36	− 61	22
			− 4.11		− 42	− 58	31
		Middle temporal gyrus	− 4.54		42	− 28	− 11
		Putamen	− 4.28		− 15	14	− 8
		Cerebellum	− 4.89		51	− 64	− 32
			− 4.67		18	− 73	− 23
			− 4.62		− 18	− 76	− 29

## Footnotes

1 We tested alternative models that did not orthogonalize parameter modulators to one another, but only to the onset regressor. The primary activation associated with SE, TE and SUP was not altered by using the alternative model (Supplementary Fig. S3).
