# Supplementary material for: The neural signature of information regularity in temporally extended event sequences
Source: Neuroimage. 2015 Feb 15;107:266–76. doi: 10.1016/j.neuroimage.2014.12.021 (PMC4306597; doi:10.1016/j.neuroimage.2014.12.021)
Supplement: Fig. S1 — Generalized linear model of RT. Within-subject GLMs included TE, SE and SUP values as predictors of single-trial RT. Coefficient estimates from individual participants were averaged. Error bars represent the standard errors across the participants. No significant effect of TE, SE or SUP on the RT at any window length (p > 0.09, Wilcoxon signed-rank test). [file mmc1.pdf]

Supporting Information

The neural systems representing information regularities in sensory events and action selections at different timescales

Jiaxiang zhang, James B Rowe

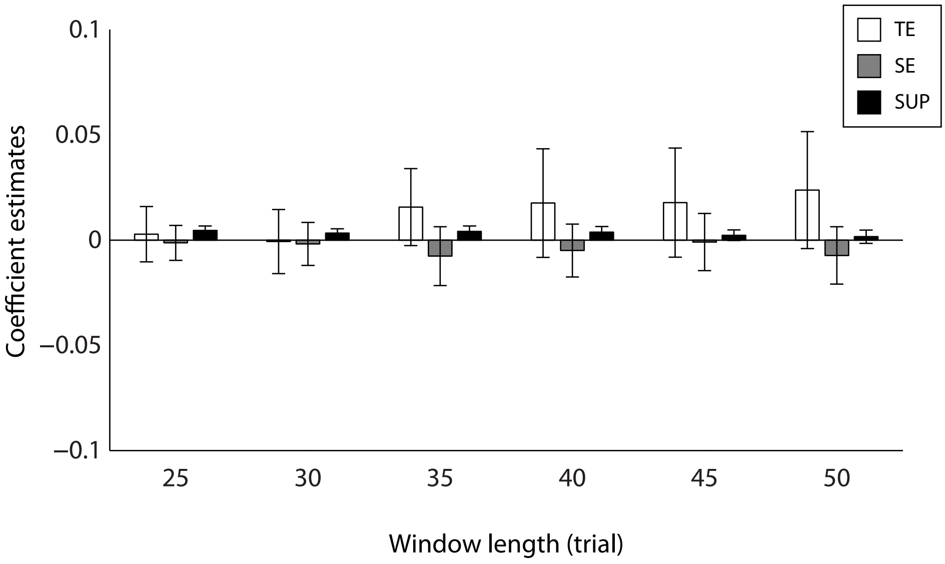

Figure S1. Generalized linear model of RT. Within-subject GLMs included TE, SE and SUP values as predictors of single-trial RT. Coefficient estimates from individual participants were averaged. Error bars represent the standard errors across the participants. No significant effect of TE, SE or SUP on the RT at any window length ($p>0.09$, Wilcoxon signed-rank test).
