# Supplementary material for: The neural signature of information regularity in temporally extended event sequences
Source: Neuroimage. 2015 Feb 15;107:266–76. doi: 10.1016/j.neuroimage.2014.12.021 (PMC4306597; doi:10.1016/j.neuroimage.2014.12.021)
Supplement: Fig. S2 — Regional BOLD response associated with trial onset. The ROIs were defined as regions with significant associations to (A) SE, (B) TE and (C) SUP as in Fig. 4. Error bars denote standard errors across participants. All ROIs had increased activity at trial onset(t(15) > 3.53, p < 0.01) except rFPO (t(15) = 2.02, p = 0.06, one-sample t-test). [file mmc2.pdf]

Supporting Information

The neural systems representing information regularities in sensory events and action selections at different timescales

Jiaxiang zhang, James B Rowe

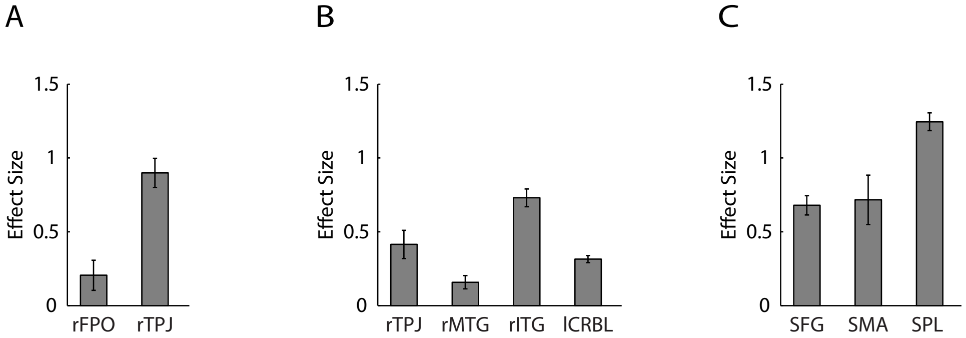

Figure S2. Regional BOLD response associated with trial onset. The ROIs were defined as regions with significant associations to (A) SE, (B) TE and (C) SUP as in Figure 4. Error bars denote standard errors across participants. All ROIs had increased activity at trial onset ($t(15)>3.53$, $p<0.01$) except rFPO ($t(15)=2.02$, $p=0.06$, one-sample t-test).
