# Supplementary material for: The neural signature of information regularity in temporally extended event sequences
Source: Neuroimage. 2015 Feb 15;107:266–76. doi: 10.1016/j.neuroimage.2014.12.021 (PMC4306597; doi:10.1016/j.neuroimage.2014.12.021)
Supplement: Fig. S3 — Brain regions showing significant responses to (A) SE, (B) TE and (C) SUP averaged across different window lengths (p<0.001 uncorrected, cluster size k>50 voxels). The model and analysis were the same as in Figure 3. The parametric regressors were only orthogonalized with respect to the trial onset regressor, and serial orthogonalization was not applied. The table below lists the statistics at peek coordinates from the model with serial orthogonalization (Figure 3) and the statistics at the same coordinates from the model without serial orthogonalization. [file mmc3.pdf]

Supporting Information

The neural systems representing information regularities in sensory events and action selections at different timescales

Jiaxiang zhang, James B Rowe

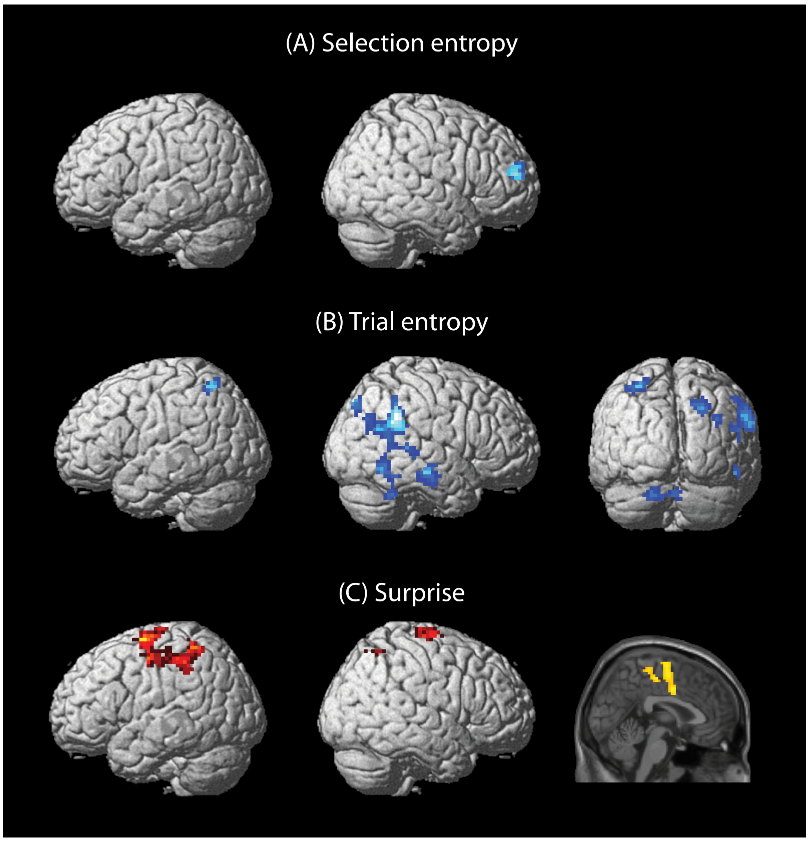

Figure S3. Brain regions showing significant responses to (A) SE, (B) TE and (C) SUP averaged across different window lengths ($p<0.001$ uncorrected, cluster size $k>50$ voxels). The model and analysis were the same as in Figure 3. The parametric regressors were only orthogonalized with respect to the trial onset regressor, and serial orthogonalization was not applied. The table below lists the statistics at peek coordinates from the model with serial orthogonalization (Figure 3) and the statistics at the same coordinates from the model without serial orthogonalization.

	Region	MNI coordinates			t-value	
		x	*y*	*z*	Serial orthogonalization	No serial orthogonalization
SE	FPC	39	53	19	4.40	4.39
	TPJ	30	-58	43	3.89	3.78
TE	CRBL	-15	-73	-29	4.71	4.59
	MTG	63	-19	-14	4.17	4.00
	ITG	48	-55	-8	4.35	4.17
	TPJ	57	-46	31	4.27	4.23
SUP	SFG	-27	-7	64	4.45	4.23
		15	-16	67	5.06	5.17
	SMA	6	-10	58	4.85	4.39
	SPL	-27	-52	55	4.94	4.77
		21	-55	55	4.00	4.54
