# Supplementary material for: The neural signature of information regularity in temporally extended event sequences
Source: Neuroimage. 2015 Feb 15;107:266–76. doi: 10.1016/j.neuroimage.2014.12.021 (PMC4306597; doi:10.1016/j.neuroimage.2014.12.021)
Supplement: Fig. S4 — Effect size plots for (A) the SE effect, and (B) the TE effect at 25-trial (blue) and 50-trial (red) window sizes. Data plotted separately for trials in which the SE or TE values were low and high in five bins (20/40/60/80/100 percentile of value range). The solid points are individual subjects' data and the open points are averaged effect size across subjects. [file mmc4.pdf]

Supporting Information

The neural systems representing information regularities in sensory events and action selections at different timescales

Jiaxiang zhang, James B Rowe

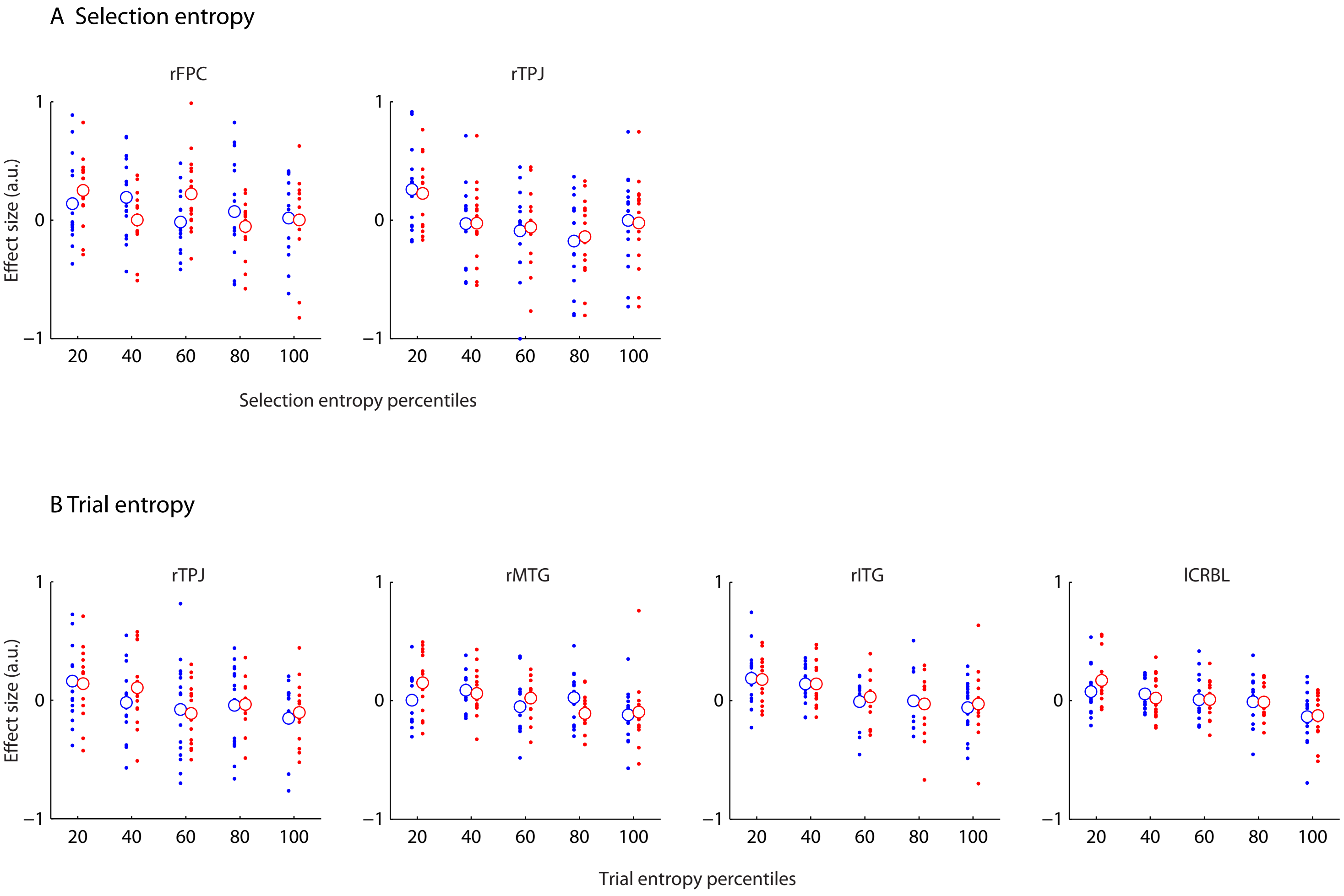

Figure S4. Effect size plots for (A) the SE effect, and (B) the TE effect at 25-trial (blue) and 50-trial (red) window sizes. Data plotted separately for trials in which the SE or TE values were low and high in five bins (20/40/60/80/100 percentile of value range). The solid points are individual subject's data and the open points are averaged effect size across subjects.
